# Progress and Challenges for Live-cell Imaging of Genomic Loci Using CRISPR-based Platforms

**DOI:** 10.1016/j.gpb.2018.10.001

**Published:** 2019-01-30

**Authors:** Xiaotian Wu, Shiqi Mao, Yachen Ying, Christopher J. Krueger, Antony K. Chen

**Affiliations:** 1 Department of Biomedical Engineering, College of Engineering, Peking University, Beijing 100871, China; 2 School of Life Sciences, Peking University, Beijing 100871, China; 3 Wallace H Coulter Department of Biomedical Engineering, Georgia Institute of Technology, Atlanta, GA 30332, USA

**Keywords:** CRISPR, Cas9, dCas9, Genomic imaging, sgRNA

## Abstract

Chromatin conformation, localization, and dynamics are crucial regulators of cellular behaviors. Although fluorescence *in situ* hybridization-based techniques have been widely utilized for investigating chromatin architectures in healthy and diseased states, the requirement for cell fixation precludes the comprehensive dynamic analysis necessary to fully understand chromatin activities. This has spurred the development and application of a variety of imaging methodologies for visualizing single chromosomal loci in the native cellular context. In this review, we describe currently-available approaches for imaging single genomic loci in cells, with special focus on clustered regularly interspaced short palindromic repeats (**CRISPR**)-based imaging approaches. In addition, we discuss some of the challenges that limit the application of CRISPR-based **genomic imaging** approaches, and potential solutions to address these challenges. We anticipate that, with continued refinement of CRISPR-based imaging techniques, significant understanding can be gained to help decipher chromatin activities and their relevance to cellular physiology and pathogenesis.

## Introduction

Over the past several decades, increasing evidence has suggested that many cellular processes, including DNA replication, DNA damage repair, and gene expression, are intimately orchestrated by genomic organization, localization, and dynamics [1], [2]. Nevertheless, our understanding of how this regulation takes place is still nascent, as existing imaging-based studies predominantly rely on fluorescence *in situ* hybridization (FISH) [3], [4], which provides high spatial but limited temporal information. Consequently, much effort has been devoted to developing strategies that enable direct visualization of individual DNA molecules in the native cellular context. Below, we briefly outline conventional approaches for imaging single genomic loci, followed by a description of clustered regularly interspaced short palindromic repeats (CRISPR)-based imaging systems, a recently-developed technology that enables live-cell imaging of single genomic loci.

## Conventional imaging techniques for labeling endogenous genomic loci

**Figure 1:**
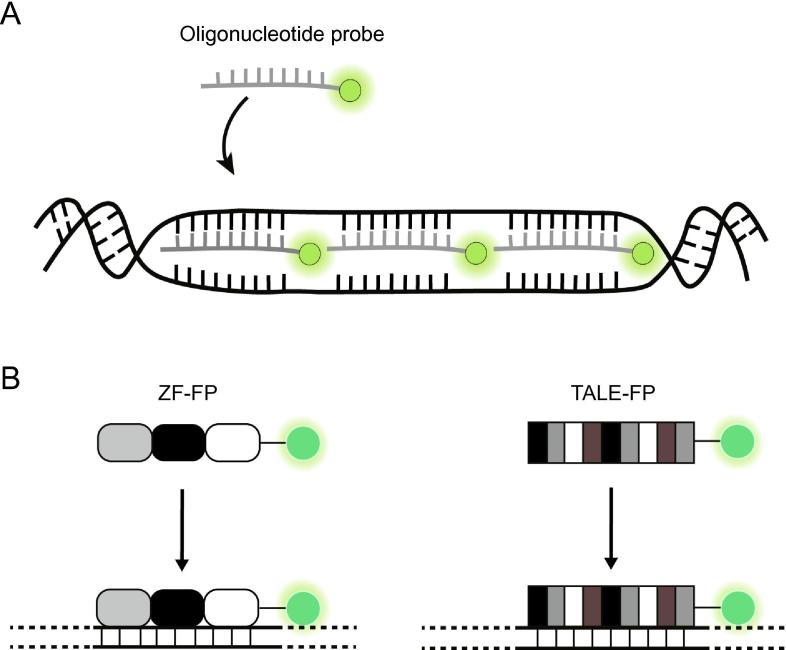
**Conventional techniques for imaging genomic loci *in situ* and in living cells** **A.** Single-molecule DNA FISH labels a genomic locus in fixed and permeabilized cells using multiple synthetic dye (light green dot)-labeled oligonucleotide probes, with probe sequences designed to hybridize with unique DNA sequences within the locus. Collective binding of the probes causes the locus to appear as a bright fluorescent spot. Note that for the probes to gain access to the target sites, the DNA duplex must be denatured. **B.** ZFs or TALEs are programmable DNA-binding proteins that can be fused to FPs (dark green dot) to enable visualization of target DNA sequences in living cells. Each ZF motif (rounded rectangle) recognizes three bases, whereas each TALE repeat (rectangle) recognizes a single base. Target sequence recognition can be programmed by combining recognition motifs. ZF, zinc finger; TALE, transcription activator-like effector; FP, fluorescent protein.

FISH has been the most commonly-used approach to map the distribution of DNA in cells [3], [4], in which synthetic dye-conjugated oligonucleotide probes are used to label DNA in fixed and permeabilized cells (Figure 1A). As the fluorescence of individual dye molecules is too faint to be detected by conventional microscopy, in order to yield single-molecule resolution, a collection of probes are used to target multiple adjacent sequences within a target locus [5]. The collective binding of multiple tagged probes to the target sequences results in a visualizable discrete bright spot indicative of a single locus. Despite the widespread application, there are several drawbacks associated with FISH. First, the need for cell fixation makes the technique cumbersome for studying chromatin dynamics. Additionally, whether the state of chromatin architecture is properly preserved after FISH processing has always been questionable, since the DNA duplex must be denatured, through use of formamide or high-temperature heating, to allow probes to hybridize to the target sequence.

Early work in live-cell genomic imaging utilized proteins capable of binding specifically to highly repetitive sequences, such as those within telomeres or centromeres [6], [7]. Accordingly, chromosome movements at the single-molecule level can be readily monitored in cells transfected with plasmids encoding repetitive sequence-binding proteins fused to fluorescent proteins (FPs). Despite these advances, the limitation of only being able to label repetitive elements precludes analysis of wider varieties of chromosome activities, since the majority of chromosomal loci are non-repetitive. More flexible approaches utilize programmable DNA-binding proteins such as zinc fingers (ZFs) [8] or transcription activator-like effectors (TALEs) [9], which are programmable to recognize specific DNA sequences (Figure 1B). However, while repetitive sequences can be readily labeled by either ZFs [10] or TALEs [11], [12], [13], [14] expressed as FP fusion proteins, only one study has successfully reported the use of such systems for imaging non-repetitive regions [15]. This may be due to the technical difficulties involved in constructing ZF or TALE expression vectors encoding multiple modules that can target multiple DNA sequences.

## CRISPR/deactivated CRISPR-associated protein 9, a powerful tool for genomic labeling

Prokaryotes possess adaptive immune systems, in which the CRISPR/CRISPR-associated (Cas) system uses small RNAs to guide a Cas nuclease to cleave invading viral or plasmid DNAs and RNAs [16]. In the Type II CRISPR system, DNA recognition and cleavage are mediated by the coordination of three components: the CRISPR RNA (crRNA), the trans-activating crRNA (tracrRNA), and the Cas9 DNA nuclease [17]. For the process to occur, crRNA and tracrRNA form an RNA duplex that recruits Cas9 to form a stable ribonucleoprotein complex [18], [19], [20]. This complex transiently binds to a short DNA sequence known as the protospacer adjacent motif (PAM). This leads to local unwinding, followed by formation of an RNA–DNA heteroduplex, if the 5′ region of the crRNA, termed spacer, is complementary to the target sequence adjacent to the PAM. Cas9 then catalyzes double-stranded breaks [21], [22], [23]. To date, the CRISPR/Cas system has been successfully adapted to serve as a versatile gene-editing platform in mammalian cells, with the majority of the applications employing a modified CRISPR/Cas system that uses only two components: Cas9 and a single guide RNA (sgRNA) that combines the functional elements of crRNA and tracrRNA [17]. Cleavage of DNA in an sgRNA-guided fashion has been shown to trigger error-prone repairs by nonhomologous end-joining [18], [19], [20], altering the sequence of a targeted gene locus.

The catalytic domains of Cas9 can be mutated to create a nuclease-deactivated form of Cas9 (dCas9), which retains the ability to interact with sgRNA and to bind to target DNA [24]. This has spurred the development of CRISPR/dCas9-based techniques for non-gene-editing applications, including noninvasive imaging of genomic loci in living cells. In these studies, researchers have modified either the dCas9 protein or sgRNA to develop DNA imaging probes that integrate FP, synthetic dye, or luminescent nanocrystal reporters in a manner that does not appear to interfere with either dCas9 binding to sgRNA or sgRNA binding to the genomic sequence. Below, we describe the progress made in each type of imaging platform and their applications in chromatin studies, followed by discussing some of the potential challenges that must be overcome in order to establish CRISPR-based imaging as a promising class of approaches for deciphering genomic activities.

### FP-based CRISPR/dCas9 systems

**Figure 2:**
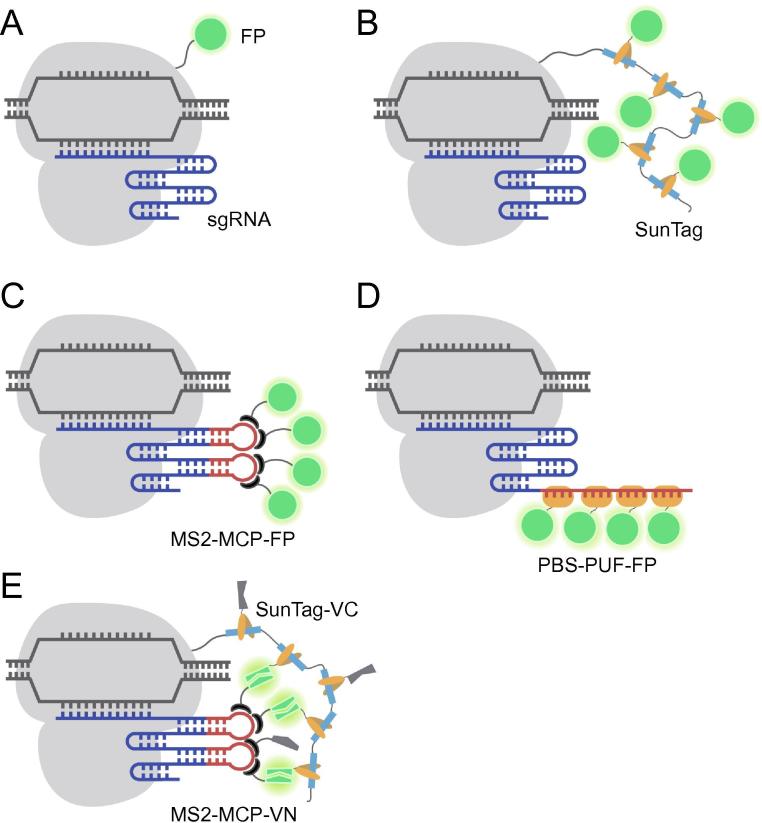
**Combining FP-based sensors and CRISPR/dCas9 for live-cell genomic labeling** In approaches that employ dCas9-FPs, dCas9 (gray) is directly fused to a single FP (**A**) or conjugated to multiple FPs through SunTag (**B**). In approaches that employ sgRNA-FPs, the sgRNA is engineered to harbor one or more copies of an RNA aptamer sequence, such as MS2, that can specifically bind to its cognate binding protein (MCP) fused to FP (**C**), or a unique target sequence (PBS) that can specifically bind to PUF family protein RNA-binding domain fused to FP (**D**). **E.** In BiFC-based approaches, dCas9 is labeled by multiple VC fragments through SunTag and sgRNA is labeled by multiple VN fragments through MS2–MCP interactions. Formation of the dCas9-sgRNA complex leads to complementation of the VC and VN fragments to form multiple copies of fluorescent Venus proteins. SunTag, supernova tagging system; MCP, MS2 coat protein; PUF, Pumilio/Fem3 mRNA-binding factor; PBS, PUF binding sequence; BiFC, bimolecular fluorescence complementation; VN, Venus N-terminal; VC, Venus C-terminal; sgRNA, single guide RNA; dCas9, deactivated CRISPR-associated protein 9.

The first use of dCas9 for genomic imaging was published by Chen et al. in 2013 [25]. In this work, the authors genetically fused dCas9 and EGFP, and demonstrated the feasibility of using dCas9-EGFP with one sgRNA to image the highly repetitive elements of the telomere, as well as to image non-repetitive regions of the *MUC4* gene through the use of an array of at least 26 different sgRNAs (Figure 2A). In a later study, Gu et al. extended the dCas9-EGFP approach to study the activity of non-repetitive regions in the enhancer and promoter of the *FGF5* gene, each using 36 unique sgRNAs [26]. Moreover, Duan et al. have demonstrated successful use of dCas9-EGFP to label highly-repetitive elements of different chromosomal loci in live mice [27]. Despite these advances, dCas9-EGFP has been observed to elicit high background signal in the nucleolus due to the tendency of the dCas9 protein to localize in the nucleolus [25], [28]. To improve gene detection, other researchers have tagged dCas9 with more FP molecules, such as through the use of the supernova tagging system (SunTag) [29], [30], [31], [32], a poly-general control noninducible 4 (GCN4) peptide scaffold that enables recruitment of up to 24 FPs through interactions between GCN4 and the single-chain variable fragment (scFv) of the antibody against GCN4 (Figure 2B). Using dCas9-SunTag, non-repetitive regions of the *MUC4* gene have been tracked continuously with only 20 different sgRNAs [30].

Alternative to dCas9-FP fusion proteins, a number of research groups have demonstrated the feasibility of imaging genomic loci using modified sgRNAs that can recruit sequence-specific RNA-binding proteins fused to FPs [33], [34], [35], [36], [37], [38], [39]. In one approach, sgRNAs have been modified to harbor multiple repeats of a unique RNA aptamer that can bind specifically to its cognate binding protein (CBP). The most widely-used aptamer is MS2, an RNA stem loop structure derived from the bacteriophage MS2 RNA virus that can bind to the MS2 coat protein (MCP) with high specificity and affinity [40], [41]. When co-expressed with MCP-FP fusion proteins, each dCas9-sgRNA complex can then be tagged by multiple FPs through MS2–MCP interactions (Figure 2C). A second approach, termed Casilio, employs the Pumilio/Fem3 mRNA-binding factor (PUF) family protein RNA-binding domain that can be programmed to bind a unique 8-mer RNA sequence (PUF binding sequence, PBS) [39]. Like MCP, PUF can also be fused to an FP while retaining its capacity to bind to PBS. Engineering sgRNA with tandem repeats of PBS makes it possible to label the dCas9-sgRNA complex with multiple FP-PUF fusion proteins (Figure 2D). Currently, both the MS2-based system and the Casilio system have enabled imaging of the highly repetitive elements within telomeres and centromeres with the use of a single sgRNA [39], [33], [34], [35], [36]. Moreover, Qin et al. showed the feasibility of the MS2-based system to image low-repeat-containing loci with a single sgRNA and non-repetitive regions of the *MUC4* gene with only 4 unique sgRNAs, each containing up to 16 MS2 aptamers [37]. While these methods show promise, Hong et al. in a recent study demonstrated that unbound dCas9-SunTag exhibits a high background signal, and sgRNAs that are extensively modified to carry large numbers of FPs have the tendency to exhibit nonspecific punctate signals that can be misinterpreted as single genomic loci [42]. The authors further showed that false positive signals could be significantly reduced in systems that deploy the bimolecular fluorescence complementation (BiFC) assay, in which the FP Venus is split into non-fluorescent amino terminal (VN) and carboxyl terminal (VC) fragments. Interaction between their respective fusion partners can bring the two fragments into close spatial proximity, leading to formation of a complete Venus protein that can then emit a signal upon excitation. In one BiFC design, dCas9 is labeled by VC through SunTag and sgRNA is labeled by VN through MS2–MCP interactions (Figure 2E). As fluorescence signal is only restored upon formation of the dCas9-VC/sgRNA-VN complex, the BiFC/dCas9-sgRNA system could illuminate specific genomic loci with higher signal-to-background compared to dCas9- or sgRNA-labeling approaches that employ whole FP reporters.

### Organic dye-based CRISPR/dCas9 systems

Compared with FPs, organic dyes are generally brighter, more photostable, and smaller in size. Therefore, a CRISPR/dCas9 imaging system that incorporates organic dye reporters could potentially benefit studies that require sensitive and continuous measurement of chromatin dynamics as compared with FP-based approaches. Nevertheless, unlike FP-based approaches in which FPs can be genetically fused to CRISPR components and expressed *in vivo* to achieve genomic labeling, attaching organic dyes to CRISPR components requires more sophisticated methods, such as bioconjugation techniques, which can be less straightforward and less specific. Additionally, many commercially available dyes cannot penetrate the cell membrane, making them difficult to use in the intracellular environment. Furthermore, biocompatibility is also a concern. Currently, three organic dye-based systems have demonstrated the feasibility for visualizing genomic loci in living cells. They include the Halo tag-based system, the RNA aptamer-based system, and the molecular beacon (MB)-based system.

**Figure 3:**
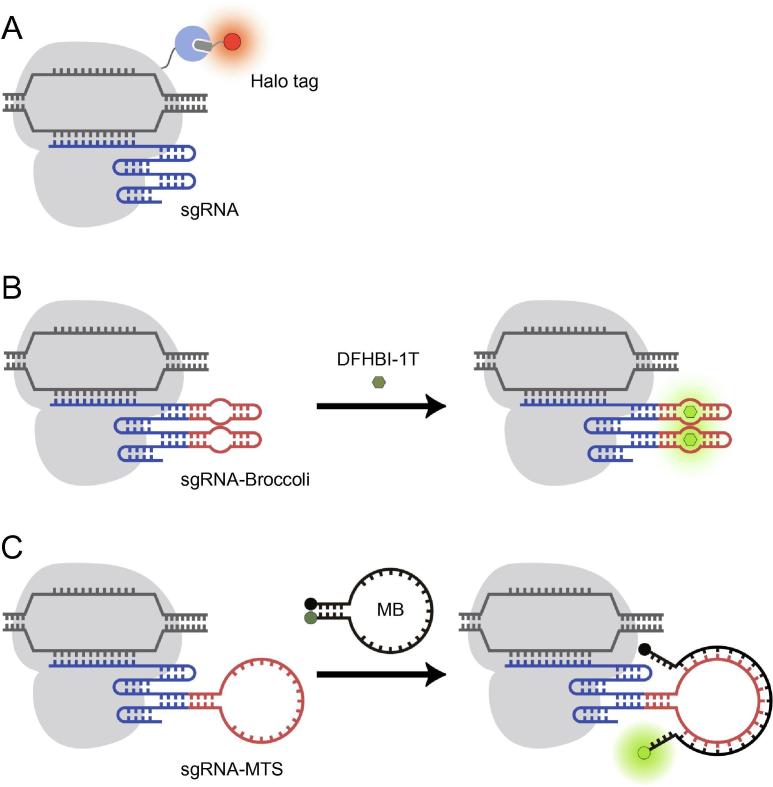
**Engineering organic dye-based CRISPR/dCas9 techniques for live-cell genomic labeling** **A.** In the dCas9-Halo tag system, dCas9 is fused to Halo tag that can bind covalently to a Halo tag ligand chemically attached to a dye of choice. **B.** In the RNA aptamer-based method, sgRNA is engineered to harbor one or more copies of an RNA aptamer (*e.g.*, Broccoli) that can bind to a cognate dye (*e.g.*, DFHBI-1T) and activate its fluorescence. **C.** In the CRISPR/MB system, sgRNA is modified to contain a unique MTS. Hybridization of the MB loop domain with the MTS separates the fluorophore (dark green dot) from the quencher (black dot), leading to restoration of MB fluorescence (light green dot). DFHBI-1T, (Z)-4-(3,5-difluoro-4-hydroxybenzylidene)-2-methyl-1-(2,2,2-trifluoroethyl)-1H-imidazol-5(4H)-one; MB, molecular beacon; MTS, MB target sequence.

In the Halo tag-based system, dCas9 has been fused to Halo tag, a mutant of the bacterial haloalkane dehalogenase enzyme that can bind covalently to a Halo tag ligand, a cell-permeable chloroalkane-based molecule that can be chemically attached to a dye of choice [43], [44]. To label a gene locus, cells were first transfected with plasmids encoding the dCas9-Halo tag fusion protein and sgRNA, followed by addition of the synthetic dye-ligand conjugate to illuminate the locus upon tag–ligand interactions (Figure 3A). The RNA aptamer-based system employs 3,5-difluoro-4-hydroxybenzylidene imidazolinone (DFHBI)-based dyes, which are activatable dyes that are well-quenched under physiological conditions but fluoresce when bound to their cognate RNA aptamers (Figure 3B) [45].

Currently, both the Halo tag-based and DFHBI-based CRISPR-labeling systems have been used to measure the nuclear dynamics and the on-target residence time of dCas9-sgRNA complexes in living cells, revealing characteristics of CRISPR system discrimination between complementary and mismatched targets [44], [45]. However, there still remains a need to improve the signal-to-background of both systems for applications where more sensitive measurements must be made. For example, in the Halo tag-based system, because unbound fluorescent ligands are unquenched, extensive washing is required to remove excess ligand from the cells, which can alter cell physiology and limit accurate assessment of chromatin dynamics. Presumably, background could be significantly reduced if a Halo tag ligand with quenchable fluorescence were used [46], but this has not yet been explored in the context of genomic imaging. In the RNA aptamer-based system, presumably owing to thermal instability and poor folding of the aptamer [47], DFHBI binding has been reported to result in fluorescence brightness comparable to FPs [48], limiting the advantages of such aptamer-based techniques for sensitive imaging of biomolecules.

Motivated by the continued need for high signal-to-background techniques, we have recently combined the CRISPR/dCas9 system with MBs, which are a class of quenchable fluorogenic oligonucleotide probes that are activated to fluoresce upon binding to complementary nucleic acid targets [49], [50]. The combined platform, termed CRISPR/MB, consists of dCas9, an MB, and an sgRNA harboring a unique MB target sequence (MTS) (Figure 3C). We showed that hybridization of MB to the sgRNA in complex with dCas9 could yield more accurate quantification and improved temporal resolution in time-lapse imaging of repetitive elements within telomere loci as compared with conventional approaches utilizing telomere repeat binding factor fused to an FP. With the flexibility in selecting fluorophore/quencher pairs to visualize genomic loci with high signal-to-background, we envision CRISPR/MB could be a promising platform for investigating chromatin activities.

### Nanoparticle-based systems for imaging genomic loci

Quantum dots (QD) are luminescent semiconductor nanoparticles, 50–100 nm in size, with brightness and photostability superior to synthetic dyes and FPs, making them excellent probes of choice in applications that require sensitive measurements, such as single-molecule imaging *in vitro*. Consequently, QD may be a promising candidate for imaging single gene loci, as its excellent optical properties may eliminate the need to target multiple loci, which may interfere with genomic functions and activities. However, as a class of synthetic nanomaterials, QDs have the limitations as mentioned above for synthetic dyes, and also have general problems pertaining to nanoparticles, thus compromising their performance *in vivo*. For example, efficient cellular delivery of QDs is difficult, owing to their large size [51]. Additionally, even if QDs are successfully delivered into cells, they are prone to entrapment in endosomes or lysosomes, forming aggregates that exhibit a high-intensity punctate staining pattern that cannot be washed away [51].

**Figure 4:**
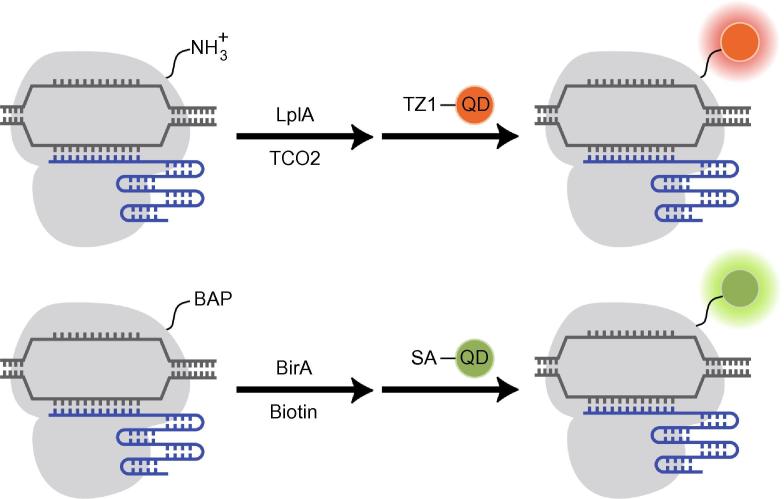
**Nanoparticle-based CRISPR/dCas9 system for live-cell genomic labeling** dCas9 can be labeled by QDs in cells through Lp1A-mediated or BirA-mediated conjugation strategies. In the former system, dCas9 is first decorated with TCO2 in the presence of Lp1A, followed by reaction with TZ1-conjugated QDs (red dot). In the latter system, dCas9 fused to a BAP tag is first biotinylated in the presence of BirA, followed by reaction with SA-conjugated QDs (green dot). QD, quantum dot; LplA, lipoic acid ligase A; BirA, biotin ligase; TCO2, trans-cyclooctene; TZ1, tetrazine; BAP, biotin acceptor peptide; SA, streptavidin.

Despite the challenges associated with nanoparticle-based approaches, in a recent study, Ma et al. used QD-labeled dCas9 and one sgRNA to image HIV-1 proviral DNA in living cells [52]. Specifically, QD was conjugated to dCas9 in the nucleus of living cells through use of lipoic acid ligase (LplA)-based or biotin/streptavidin-based methods (Figure 4). For the former method, dCas9 fused to the acceptor peptide of LplA was ligated to trans-cyclooctene (TCO2) in the presence of LplA in cells. Tetrazine-modified QDs were then transfected into the cells, labeling the dCas9 via Diels–Alder cycloaddition [52]. For the latter method, dCas9 was fused to a 15-amino acid peptide (termed biotin acceptor peptide (BAP) tag) that can then be biotinylated in the presence of biotin ligase in cells. This was followed by transfection of streptavidin-modified QDs to label the dCas9 protein [52].

### Multiple-color labeling of genomic loci

**Figure 5:**
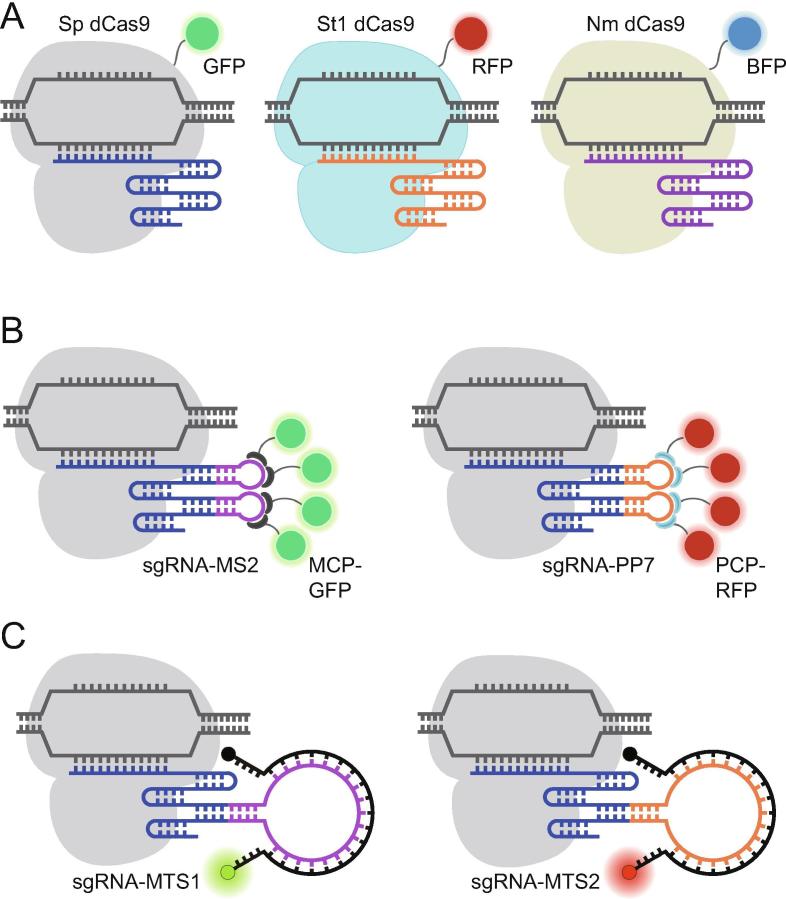
**CRISPR/dCas9 techniques for simultaneous imaging of multiple genomic loci in living cells** Simultaneous imaging of multiple genomic loci has been achieved through the use of dCas9 orthologs including Sp dCas9 (gray), St1 dCas9 (ligh blue), and Nm dCas9 (light yellow) that recognize different PAM sequences and sgRNA scaffolds (**A**), orthogonal RNA aptamer/CBP systems (MS2/MCP and PP7/PCP) in combination with a dCas9 species (**B**), or CRISPR/MB systems with optically-distinct MBs that target orthogonal MTSs in combination with a dCas9 species (**C**). BFP, blue flurescent protein; PAM, protospacer adjacent motif; CBP, cognate binding protein; MCP, MS2 coat protein; PCP, PP7 coat protein.

The ability to simultaneously illuminate multiple unique genomic elements in living cells is crucial for comprehensive understanding of dynamic regulation of genomic architectures. To date, most of the aforementioned CRISPR/dCas9 imaging methods have been extended for multiplex genomic imaging in living cells [53], [33], [34], [35], [36]. For example, in studies employing dCas9-FP, up to 3 genomic loci have been visualized simultaneously using three dCas9 orthologs, with each ortholog derived from a different bacteria species and capable of recognizing a unique PAM sequence and a unique sgRNA scaffold [53] (Figure 5A). By co-transfecting plasmid constructs encoding each pair of dCas9-FP and its cognate sgRNA, the distance between loci on the same chromosome or different chromosomes was measured. In methods that rely on RNA–protein interactions, such as aptamer-based systems, dual-color imaging has been achieved using a single dCas9 species and two sgRNAs harboring orthogonal RNA aptamers that can recognize cognate effectors tagged by opticallydistinct FPs [33], [35], [36]. The most commonly used orthogonal aptamers are MS2 and PP7, RNA stem loop structures that are derived from bacteriophage MS2 and PP7 RNA viruses, respectively [54]. Similar to MS2, PP7 binds to the PP7 bacteriophage coat protein (PCP) with high specificity and affinity (Figure 5B) [54]. Since the MS2/MCP system and the PP7/PCP system exhibit mutually-exclusive reactivity, different loci could be labeled simultaneously. Using this approach, relative distances between different chromosomes and between regions within one chromosome were tracked throughout each stage of the cell cycle [33], [35]. Furthermore, CRISPRainbow uses sgRNAs genetically modified to carry up to 3 types of RNA aptamers (MS2, PP7, and BoxB) in a combinatorial fashion [34]. When co-expressed with their CBPs fused with optically-distinct FPs, each sgRNA scaffold can then be labeled by one or more types of FPs through specific aptamer–protein interactions. Overlaying the fluorescence signals enables simultaneous live-cell visualization of up to 6 different chromosomal loci, revealing large differences in the dynamic properties of different chromosomes and of different loci within a chromosome. Finally, CRISPR/MB can incorporate a wide variety of fluorophores and MTS sequences. With the use of a second unique MTS sequence and an optically-distinct MB probe (Figure 5C), telomere and centromere loci were shown to exhibit similar dynamic behaviors [49].

## Overcoming challenges in CRISPR-based imaging

Despite great progress made in the CRISPR-based imaging field, many challenges still remain to be overcome before the technology can truly be useful in furthering our understanding of the role of chromatin activities in health and disease. Below, we outline several existing challenges and provide possible solutions to these challenges.

### Off-target binding and target site availability

*Streptococcus pyogenes* Cas9 (SpCas9), the most commonly used Cas9 variant, has a relatively simple PAM requirement (NGG), which gives flexibility in target site selection, but associated high-frequency off-target binding may lead to false-positive signals when used for imaging [55], [56]. To address this concern, Cas9 orthologs from different bacterial species with varying PAM availabilities might be used [57], [58]. For example, *Neisseria meningitides* Cas9 (NmCas9) has a longer PAM sequence, which limits target site selection but has been reported to reduce off-target binding [57]. Alternatively, other recently discovered SpCas9 variants, such as enhanced-specificity SpCas9 (eSpCas9) and expanded PAM SpCas9 variant (xCas9) [59], [60], which have been demonstrated to possess lower off-target activity and/or broader PAM compatibility compared with wild type SpCas9, might be modified to enable imaging of genomic loci with enhanced specificity and a wider DNA target region selection. Another approach could involve the use of other Cas proteins, such as Cas12a (Cpf1), which exhibits reduced mismatch tolerance as compared with SpCas9 [61], [62].

### Target accessibility

Even use of a hypothetical dCas9 species devoid of off-target effect and with unrestricted PAM specificity may not enable visualization of all genomic loci, as target DNA regions may be bound by cognate DNA-binding proteins, making them inaccessible to dCas9/sgRNA labeling. Similarly, as DNA is a highly-structured molecule, regions with high levels of topological complexity may also be inaccessible to dCas9/sgRNA labeling. At present, it is still not possible to determine the complete conformation of DNA and its variations in a spatial–temporal manner. To improve target selection, ChIP sequencing (ChIP-seq) could unravel regions that are highly prone to protein binding. Additionally, chromosome conformation capture (3C) could provide further insights into 3-dimensional chromatin organization.

### Target selectivity

When using the SpCas9 system, the spacer sequence and spacer length may influence the efficiency of target site binding. For example, binding of sgRNA to the non-transcribed strand appears to be more effective than to the transcribed strand [63]. This can potentially hamper imaging of genomic loci containing insufficient numbers of PAM sequences in the non-transcribed strand. Additionally, Cas9 preferentially binds to sgRNAs containing purines in the last four nucleotides of the spacer [63]. Furthermore, sgRNA with a moderate number of GC nucleotides shows higher efficiency for target binding [63]. Presumably, the SpCas9 system may be modified to increase target selectivity.

### Background fluorescence

To increase signal-to-background ratio, much effort has been devoted to increasing signal via fluorescent labeling of either dCas9 or sgRNA. This inevitably raises the background signals due to the presence of free fluorescently-tagged dCas9, sgRNA, or dCas9-sgRNA complexes unbound to the target site. While optimization of transfection conditions has been a means to reduce background, it is known that transfection efficacy is difficult to control and can vary significantly from cell to cell. Although one potential strategy to gain better control of transfection is through generation of stable cell lines, or the use of lentivirus-based delivery methods, these operations can potentially alter cell physiology, thus defeating the purpose of noninvasive endogenous imaging. Presumably, reducing background signal may require the implementation of more sophisticated imaging methods, such as fluorescence resonance energy transfer (FRET), which has been used for background-free imaging of RNA [64] and proteins [65], [66], [67].

### Imaging non-repetitive sequences

Compared to imaging of repetitive elements, which requires only one sgRNA, visualizing non-repetitive elements is more difficult owing to the need to use multiple unique sgRNAs. In early work [25], [37], multiple constructs, each encoding a unique sgRNA, were transfected into cells using rigorously optimized transfection conditions. Recent approaches involve the use of multiple sgRNAs cloned into a single plasmid constructed by chimeric array of gRNA oligos (CARGO) [26] or Golden Gate Assembly [30], which simplifies transfection procedures while improving transfection efficiency. Despite these advances, simultaneous co-expression of multiple different sgRNA species in one cell can still be difficult, because the transcription rate of RNAs often exhibit pulsatile variations [68], [69], [70], [71], [72]. As a result, production of the multiple sgRNAs may be “out of sync” with one another. To increase co-expression of different sgRNAs, one potential strategy could involve engineering an expression plasmid encoding different sgRNAs in one transcript, with every two sgRNAs linked by a substrate that can be excised by RNases. One candidate for such a substrate is tRNA, which has been used to liberate multiple individual *MUC4*- and *MUC1*-targeting sgRNAs from a single RNA transcript [73]. It should also be noted that even if all of the different sgRNAs could be expressed simultaneously, imaging of non-repetitive regions could still be challenging, since it is possible that different sgRNAs can compete with each other for binding to dCas9. Using multiple dCas9 orthologs may be a potential strategy to reduce the competition among different sgRNAs.

## Concluding remarks

Since the first successful repurposing of the CRISPR system to visualize genomic activities in living cells, numerous groups have been working to develop derivative systems with improved optical characteristics, dCas9/sgRNA properties, and labeling strategies, with the overall goal of enhancing the sensitivity and reliability of genomic detection. A robust CRISPR-based imaging system could aid in revealing new insights into how chromatin structure and dynamics influence cell function in normal and disease states, information which is not easily attainable by current biochemistry-based tools. For example, to decipher genome organizations, specific loci that are highly prone to intra-chromosomal and inter-chromosomal interactions could be visualized over time with high spatiotemporal resolutions. Additionally, to study epigenetic regulation, spatial and temporal coupling of gene expression to chromatin conformation could also be further explored. Furthermore, in the context of RNA/DNA biology, a robust chromatin imaging system could be combined with an RNA imaging platform, such as an RNA-targeting Cas9 platform [74], [75], to simultaneously visualize chromatin and long non-coding RNAs, which are increasingly understood to play critical roles in the regulation of chromosomal stability and activities [76], [77]. Last but not least, a robust system could also be useful in molecular diagnostics of human diseases, including cancer and neurodegenerative diseases, which have been linked to chromatin dysregulation [78], [79]. We should also emphasize that besides mammalian cells, CRISPR-based imaging technology has also been utilized to illuminate DNA in numerous other species, such as yeast and plant cells [80], [81]. Thus, with continued improvement in the CRISPR-based imaging field, we envision that the technology could facilitate studies of genomic activities in different biological contexts.

## Competing interests

The authors have declared no competing interests.
